# (1*S*,2*R*,3*R*,6*R*,7*S*,8*R*,10*S*,11*S*)-13-{[4-(4-Chloro­phen­yl)piperazin-1-yl]meth­yl}-10-hy­droxy-4,9-dimethyl-3,8,15-trioxatetra­cyclo­[10.3.0.0^2,4^.0^7,9^]penta­decan-14-one

**DOI:** 10.1107/S1600536812011816

**Published:** 2012-03-24

**Authors:** Mohamed Moumou, Ahmed Benharref, Lahcen El Ammari, Mina Adil, Moha Berraho

**Affiliations:** a Laboratoire de Chimie Bioorganique et Analytique, URAC 22, BP 146, FSTM, Université Hassan II, Mohammedia–Casablanca 20810 Mohammedia, Morocco; b Laboratoire de Chimie Biomoléculaire, Substances Naturelles et Réactivité, URAC 16, Faculté des Sciences Semlalia, BP 2390, Bd My Abdellah,40000 Marrakech, Morocco; c Laboratoire de Chimie du Solide Applique’e, Faculté des Sciences, Avenue Ibn Battouta, BP 1014 Rabat, Morocco

## Abstract

The title compound, C_25_H_33_ClN_2_O_5_, was synthesized from 9α-hy­droxy­parthenolide (9α-hy­droxy-4,8-dimethyl-12-methyl­ene-3,14-dioxatricyclo­[9.3.0.0^2,4^]tetra­dec-7-en-13-one), which was isolated from the chloro­form extract of the aerial parts of *Anvillea radiata*. The mol­ecule is built up from fused five- and ten-membered rings with two additional ep­oxy ring systems and a chloro­phenyl­piperazine group as a substituent. The ten-membered ring adopts an approximate chair–chair conformation, while the piperazine ring displays a chair conformation and the five-membered ring shows an envelope conformation with the C atom closest to the hy­droxy group forming the flap. The mol­ecular conformation is stabilized by an intra­molecular O—H⋯N hydrogen bond between the hy­droxy group and a piperazine N atom. The crystal structure is stabilized by weak C—H⋯O inter­actions.

## Related literature

For background to the medicinal uses of the plant *Anvillea adiata*, see: El Hassany *et al.* (2004 ▶); Qureshi *et al.* (1990 ▶). For the reactivity of this sesquiterpene, see: Hwang *et al.* (2006 ▶); Neukirch *et al.* (2003 ▶); Neelakantan *et al.* (2009 ▶); Castaneda-Acosta *et al.* (1997 ▶). For ring puckering parameters, see: Cremer & Pople (1975 ▶). For the synthetic procedure, see: Moumou *et al.* (2010 ▶).
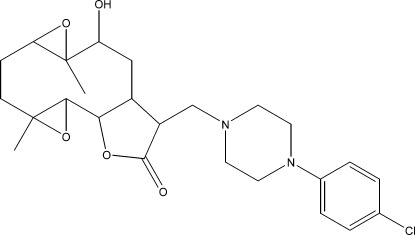


## Experimental

### 

#### Crystal data

C_25_H_33_ClN_2_O_5_
*M* *_r_* = 476.98
Orthorhombic, 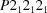

*a* = 8.0138 (3) Å
*b* = 10.7218 (5) Å
*c* = 28.0174 (13) Å
*V* = 2407.32 (18) Å^3^
*Z* = 4
Mo *K*α radiation
μ = 0.20 mm^−1^
*T* = 296 K
0.33 × 0.17 × 0.04 mm

#### Data collection

Agilent Xcalibur Sapphire1 long nozzle diffractometer
13518 measured reflections
4328 independent reflections
3315 reflections with *I* > 2σ(*I*)
*R*_int_ = 0.030

#### Refinement

*R*[*F* ^2^ > 2σ(*F* ^2^)] = 0.041
*wR*(*F* ^2^) = 0.102
*S* = 1.03
4328 reflections
301 parameters
H-atom parameters constrained
Δρ_max_ = 0.22 e Å^−3^
Δρ_min_ = −0.21 e Å^−3^
Absolute structure: Flack (1983 ▶), 1836 Friedel pairs
Flack parameter: 0.03 (13)

### 

Data collection: *CrysAlis PRO* (Agilent, 2010 ▶); cell refinement: *CrysAlis PRO*; data reduction: *CrysAlis PRO*; program(s) used to solve structure: *SHELXS97* (Sheldrick,2008 ▶); program(s) used to refine structure: *SHELXL97* (Sheldrick,2008 ▶); molecular graphics: *ORTEP-3 for Windows* (Farrugia, 1997 ▶) and *PLATON* (Spek, 2009 ▶); software used to prepare material for publication: *WinGX* (Farrugia, 1999 ▶).

## Supplementary Material

Crystal structure: contains datablock(s) I, global. DOI: 10.1107/S1600536812011816/bt5834sup1.cif

Structure factors: contains datablock(s) I. DOI: 10.1107/S1600536812011816/bt5834Isup2.hkl

Supplementary material file. DOI: 10.1107/S1600536812011816/bt5834Isup3.cml

Additional supplementary materials:  crystallographic information; 3D view; checkCIF report

## Figures and Tables

**Table 1:** Hydrogen-bond geometry (Å, °)

*D*—H⋯*A*	*D*—H	H⋯*A*	*D*⋯*A*	*D*—H⋯*A*
O2—H2*A*⋯N1	0.82	2.14	2.943 (2)	166
C1—H1⋯O4^i^	0.98	2.37	3.271 (3)	152
C10—H10⋯O1^ii^	0.98	2.41	3.329 (3)	153

Symmetry codes: (i) 
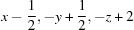
; (ii) 
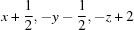
.

**Fig. 1.**
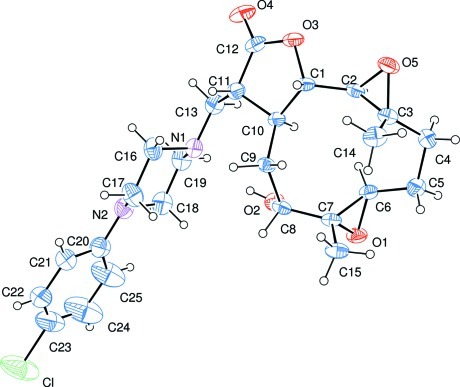
: Molecular structure of the title compound with the atom-labelling scheme. Displacement ellipsoids are drawn at the 30% probability level. H atoms are represented as small spheres of arbitrary radii.

**Fig. 2.**
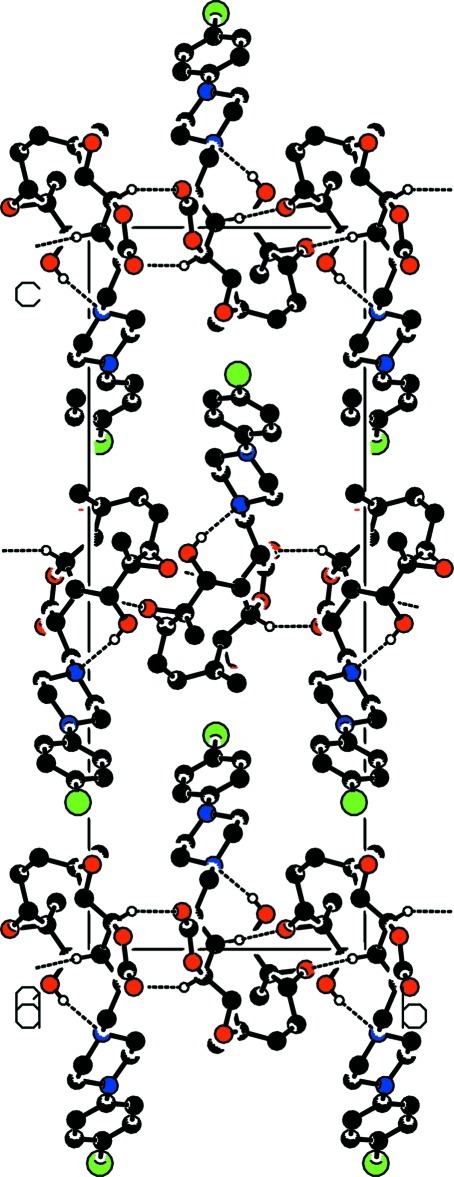
: Packing view showing the C–H···O and O–H···N hydrogen bonds as dashed lines. H atoms not involved in hydrogen bonding have been omitted for clarity.

## supplementary crystallographic information

### Comment

Our work lies within the framework of the valorization of medicinals plants and concerning the Anvillea radiata. The main constituent of the chloroform extract of aerial parts of this plant is 9α-hydroxypartenolide (El Hassany *et al.*, 2004). The reactivity of this sesquiterpene lactone and its derivatives has been the subject of several studies (Castaneda-Acosta *et al.*,1997; Neukirch *et al.*, 2003; Hwang *et al.*, 2006; Neelakantan *et al.*, 2009). In order to prepare products with a high added value that can be used in the pharmacological and cosmetics industry. In this context, we have synthesized from 9α-hydroxyparthenolide the 6β,7α-epoxy-9apha hydoxypartenolide(9α-hydroxy-4,8-dimethyl-12- methylen-3,14-dioxa-tricyclo[9.3.0.0^2^,^4^]tetradec-7-en-13-one) (Moumou *et al.*, 2010). This epoxy-hydroxypartenolide treated with one equivalent of 1-(4-chlorophenyl)-piperazine gives the title compound with a yield of 80%. Its crystal structure is reported herein. The molecule contains a fused ring system and the chlorophenyl-piperazine group as a substituent to the lactone ring. The molecular structure (Fig.1) shows that the lactone ring adopts an envelope conformation, as indicated by Cremer & Pople (1975) puckering parameters Q = 0.309 (2) Å and φ = 259.0 (4)°. The ten-membered ring displays an approximate chair-chair conformation, while the piperazine ring has a chair conformation with Q_T_ = 0.563 (3) Å, θ = 172.4 (3)° and φ2 = 187 (2)°. In the crystal structure, the molecules are linked by C—H···O intermolecular hydrogen bonds into chains along the *b* axis (Table 1, Fig. 2). In addition, an intramolecular O—H···N hydrogen bond is also observed. Owing to the presence of a Cl atom, the absolute configuration could be fully confirmed, by refining the Flack parameter (Flack, 1983) as C1(S), C2(*R*) C3(*R*), C6(*R*), C7(S), C8(*R*), C10(S), C11(S).

### Experimental

The mixture of 6β,7α-epoxy-9α-hydoxypartenolide (9α-hydroxy-4,8- dimethyl-12-methylen-3,14-dioxa-tricyclo[9.3.0.0^2^,^4^] tetradec-7-en-13-one)(700 mg, 2,5 mmol) and one equivalent of 1-(4- chlorophenyl)-piperazine)in EtOH (20 ml) was stirred for twelve hours at room temperature. Then the reaction was stopped by adding water(10 ml) and extracted three times with ethyl acetate (3 *x* 20 ml). The combined organic layers were dried over anhydrous MgSO_4_, filtered and concentrated under vacuum to give 953 mg (2 mmol) of the title compound (yield: 80%). Recrystallization was performed from ethyl acetate.

### Refinement

Reflections (0 0 2), (1 0 1) and (0 1 1) were obstructed by the beam stop and were omitted from the refinement. All H atoms were fixed geometrically and treated as riding with O—H = 0.82 Å, C—H = 0.96 Å (methyl), 0.97 Å (methylene), 0. 98 Å (methine) with *U*_iso_(H) = 1.2*U*_eq_ (methylene, methine) or *U*_iso_(H) = 1.5*U*_eq_ (methyl, OH).

### Crystal data

**Table:**

C_25_H_33_ClN_2_O_5_	*F*(000) = 1016
*M**_r_* = 476.98	*D*_x_ = 1.316 Mg m^−^^3^
Orthorhombic, *P*2_1_2_1_2_1_	Mo *K*α radiation, λ = 0.71073 Å
Hall symbol: P 2ac 2ab	Cell parameters from 13518 reflections
*a* = 8.0138 (3) Å	θ = 2.4–25.2°
*b* = 10.7218 (5) Å	µ = 0.20 mm^−^^1^
*c* = 28.0174 (13) Å	*T* = 296 K
*V* = 2407.32 (18) Å^3^	Patelet, colourless
*Z* = 4	0.33 × 0.17 × 0.04 mm

### Data collection

**Table:**

Agilent Xcalibur Sapphire1 long nozzle diffractometer	3315 reflections with *I* > 2σ(*I*)
Radiation source: fine-focus sealed tube	*R*_int_ = 0.030
Graphite monochromator	θ_max_ = 25.2°, θ_min_ = 2.4°
Detector resolution: 8.2632 pixels mm^-1^	*h* = −9→7
ω scans	*k* = −12→12
13518 measured reflections	*l* = −31→33
4328 independent reflections	

### Refinement

**Table:**

Refinement on *F*^2^	Secondary atom site location: difference Fourier map
Least-squares matrix: full	Hydrogen site location: inferred from neighbouring sites
*R*[*F*^2^ > 2σ(*F*^2^)] = 0.041	H-atom parameters constrained
*wR*(*F*^2^) = 0.102	*w* = 1/[σ^2^(*F*_o_^2^) + (0.0489*P*)^2^ + 0.3159*P*] where *P* = (*F*_o_^2^ + 2*F*_c_^2^)/3
*S* = 1.03	(Δ/σ)_max_ = 0.001
4328 reflections	Δρ_max_ = 0.22 e Å^−^^3^
301 parameters	Δρ_min_ = −0.21 e Å^−^^3^
0 restraints	Absolute structure: Flack (1983), 1836 Friedel pairs
Primary atom site location: structure-invariant direct methods	Flack parameter: 0.03 (13)

### Special details

**Table:**

Geometry. All s.u.'s (except the s.u. in the dihedral angle between two l.s. planes) are estimated using the full covariance matrix. The cell s.u.'s are taken into account individually in the estimation of s.u.'s in distances, angles and torsion angles; correlations between s.u.'s in cell parameters are only used when they are defined by crystal symmetry. An approximate (isotropic) treatment of cell s.u.'s is used for estimating s.u.'s involving l.s. planes.
Refinement. Refinement of *F*^2^ against ALL reflections. The weighted *R*-factor *wR* and goodness of fit *S* are based on *F*^2^, conventional *R*-factors *R* are based on *F*, with *F* set to zero for negative *F*^2^. The threshold expression of *F*^2^ > 2σ(*F*^2^) is used only for calculating *R*-factors(gt) *etc*. and is not relevant to the choice of reflections for refinement. *R*-factors based on *F*^2^ are statistically about twice as large as those based on *F*, and *R*- factors based on ALL data will be even larger.

### Fractional atomic coordinates and isotropic or equivalent isotropic displacement parameters (Å^2^)

**Table:**

	*x*	*y*	*z*	*U*_iso_*/*U*_eq_	
C1	0.5658 (3)	0.08524 (19)	1.03681 (8)	0.0420 (6)	
H1	0.5100	0.1552	1.0527	0.050*	
C2	0.5983 (3)	−0.0172 (2)	1.07076 (8)	0.0417 (5)	
H2	0.6496	−0.0907	1.0561	0.050*	
C3	0.4995 (3)	−0.0452 (2)	1.11324 (8)	0.0486 (6)	
C4	0.4939 (3)	−0.1805 (2)	1.12703 (9)	0.0570 (7)	
H4A	0.4843	−0.1867	1.1615	0.068*	
H4B	0.5981	−0.2197	1.1178	0.068*	
C5	0.3493 (3)	−0.2521 (2)	1.10412 (9)	0.0578 (7)	
H5A	0.3715	−0.3408	1.1065	0.069*	
H5B	0.2480	−0.2349	1.1219	0.069*	
C6	0.3212 (3)	−0.2194 (2)	1.05269 (8)	0.0451 (5)	
H6	0.4234	−0.2069	1.0341	0.054*	
C7	0.1731 (3)	−0.1534 (2)	1.03400 (8)	0.0435 (5)	
C8	0.1828 (3)	−0.0735 (2)	0.98945 (8)	0.0436 (5)	
H8	0.0683	−0.0523	0.9803	0.052*	
C9	0.2755 (2)	0.04992 (19)	0.99890 (8)	0.0394 (5)	
H9A	0.2505	0.0772	1.0311	0.047*	
H9B	0.2329	0.1128	0.9772	0.047*	
C10	0.4669 (2)	0.04236 (19)	0.99307 (7)	0.0387 (5)	
H10	0.4957	−0.0450	0.9870	0.046*	
C11	0.5445 (3)	0.1206 (2)	0.95335 (9)	0.0466 (6)	
H11	0.4888	0.2019	0.9524	0.056*	
C12	0.7203 (3)	0.1384 (2)	0.97059 (10)	0.0530 (6)	
C13	0.5433 (3)	0.0646 (3)	0.90349 (9)	0.0570 (6)	
H13A	0.6072	0.1181	0.8824	0.068*	
H13B	0.5983	−0.0160	0.9044	0.068*	
C14	0.3600 (3)	0.0359 (3)	1.13124 (9)	0.0605 (7)	
H14A	0.3633	0.0388	1.1655	0.091*	
H14B	0.2550	0.0020	1.1211	0.091*	
H14C	0.3727	0.1187	1.1187	0.091*	
C15	0.0266 (3)	−0.1177 (3)	1.06488 (10)	0.0604 (7)	
H15A	−0.0753	−0.1407	1.0492	0.091*	
H15B	0.0276	−0.0292	1.0702	0.091*	
H15C	0.0341	−0.1603	1.0949	0.091*	
C16	0.2978 (4)	0.1685 (2)	0.87270 (10)	0.0625 (7)	
H16A	0.3649	0.2123	0.8493	0.075*	
H16B	0.2932	0.2192	0.9013	0.075*	
C17	0.1245 (4)	0.1503 (3)	0.85354 (10)	0.0708 (8)	
H17A	0.0556	0.1119	0.8779	0.085*	
H17B	0.0765	0.2309	0.8459	0.085*	
C18	0.2143 (4)	−0.0440 (3)	0.81888 (10)	0.0701 (8)	
H18A	0.2250	−0.0878	0.7887	0.084*	
H18B	0.1511	−0.0966	0.8405	0.084*	
C19	0.3848 (4)	−0.0219 (3)	0.83936 (9)	0.0638 (7)	
H19A	0.4387	−0.1014	0.8453	0.077*	
H19B	0.4520	0.0237	0.8165	0.077*	
C20	−0.0295 (4)	0.0626 (3)	0.78687 (10)	0.0725 (8)	
C21	−0.1371 (5)	0.1627 (4)	0.78375 (14)	0.1020 (12)	
H21	−0.1106	0.2367	0.7993	0.122*	
C22	−0.2840 (5)	0.1548 (5)	0.75781 (18)	0.1239 (17)	
H22	−0.3552	0.2232	0.7564	0.149*	
C23	−0.3247 (5)	0.0485 (8)	0.73460 (13)	0.1231 (19)	
C24	−0.2246 (7)	−0.0525 (8)	0.73785 (16)	0.157 (2)	
H24	−0.2548	−0.1264	0.7228	0.188*	
C25	−0.0770 (5)	−0.0464 (5)	0.76352 (14)	0.1235 (16)	
H25	−0.0085	−0.1163	0.7652	0.148*	
N1	0.3753 (2)	0.04868 (18)	0.88377 (6)	0.0494 (5)	
N2	0.1238 (3)	0.0725 (2)	0.81120 (7)	0.0628 (6)	
O1	0.1903 (2)	−0.28639 (14)	1.02786 (6)	0.0532 (4)	
O2	0.2538 (2)	−0.14084 (14)	0.95132 (6)	0.0524 (4)	
H2A	0.2752	−0.0930	0.9293	0.079*	
O3	0.72935 (19)	0.12359 (15)	1.01782 (6)	0.0526 (4)	
O4	0.8427 (2)	0.16250 (18)	0.94779 (8)	0.0750 (6)	
O5	0.6623 (2)	0.01156 (16)	1.11736 (6)	0.0582 (5)	
Cl	−0.51185 (15)	0.0396 (2)	0.70351 (4)	0.1995 (9)	

### Atomic displacement parameters (Å^2^)

**Table:**

	*U*^11^	*U*^22^	*U*^33^	*U*^12^	*U*^13^	*U*^23^
C1	0.0249 (11)	0.0402 (12)	0.0608 (15)	−0.0039 (9)	0.0027 (10)	−0.0073 (10)
C2	0.0270 (11)	0.0431 (12)	0.0550 (13)	−0.0006 (10)	−0.0066 (10)	−0.0054 (10)
C3	0.0383 (13)	0.0548 (14)	0.0526 (13)	−0.0048 (12)	−0.0054 (11)	−0.0063 (12)
C4	0.0538 (16)	0.0640 (17)	0.0531 (15)	−0.0004 (13)	−0.0068 (12)	0.0055 (12)
C5	0.0558 (17)	0.0509 (15)	0.0668 (17)	−0.0033 (12)	−0.0025 (13)	0.0080 (12)
C6	0.0370 (13)	0.0395 (11)	0.0587 (15)	−0.0069 (11)	0.0010 (11)	−0.0022 (11)
C7	0.0276 (12)	0.0436 (12)	0.0592 (14)	−0.0060 (10)	−0.0002 (10)	−0.0094 (11)
C8	0.0262 (11)	0.0475 (12)	0.0571 (14)	0.0011 (10)	−0.0054 (10)	−0.0073 (11)
C9	0.0287 (11)	0.0375 (10)	0.0519 (12)	0.0045 (10)	−0.0023 (9)	−0.0048 (10)
C10	0.0321 (11)	0.0312 (10)	0.0529 (13)	0.0003 (9)	0.0017 (10)	−0.0025 (10)
C11	0.0407 (13)	0.0394 (11)	0.0596 (14)	0.0009 (11)	0.0045 (11)	0.0011 (11)
C12	0.0449 (15)	0.0371 (12)	0.0769 (19)	−0.0073 (11)	0.0137 (14)	−0.0016 (12)
C13	0.0545 (16)	0.0561 (15)	0.0603 (15)	0.0075 (13)	0.0122 (12)	0.0010 (13)
C14	0.0567 (16)	0.0644 (16)	0.0605 (15)	−0.0039 (15)	0.0105 (13)	−0.0146 (13)
C15	0.0323 (13)	0.0733 (17)	0.0756 (18)	−0.0046 (13)	0.0082 (13)	−0.0063 (14)
C16	0.074 (2)	0.0525 (15)	0.0609 (16)	0.0130 (14)	−0.0042 (14)	−0.0013 (12)
C17	0.0699 (19)	0.0800 (19)	0.0627 (17)	0.0234 (17)	−0.0014 (15)	−0.0092 (15)
C18	0.091 (2)	0.0634 (16)	0.0558 (16)	0.0037 (17)	0.0012 (15)	−0.0100 (14)
C19	0.078 (2)	0.0597 (16)	0.0538 (15)	0.0120 (15)	0.0097 (14)	−0.0076 (13)
C20	0.070 (2)	0.107 (2)	0.0399 (14)	−0.011 (2)	0.0070 (14)	0.0107 (16)
C21	0.078 (3)	0.103 (3)	0.125 (3)	−0.015 (2)	−0.027 (2)	0.042 (2)
C22	0.078 (3)	0.161 (4)	0.132 (4)	−0.029 (3)	−0.026 (3)	0.074 (3)
C23	0.072 (3)	0.246 (6)	0.051 (2)	−0.036 (4)	−0.0035 (18)	0.020 (3)
C24	0.107 (4)	0.273 (8)	0.090 (3)	−0.009 (5)	−0.012 (3)	−0.086 (4)
C25	0.095 (3)	0.184 (4)	0.091 (3)	0.010 (3)	−0.011 (2)	−0.073 (3)
N1	0.0549 (13)	0.0464 (10)	0.0470 (11)	0.0071 (10)	0.0056 (10)	−0.0016 (9)
N2	0.0683 (16)	0.0730 (15)	0.0470 (12)	0.0009 (12)	0.0021 (11)	0.0035 (11)
O1	0.0456 (10)	0.0396 (8)	0.0743 (11)	−0.0113 (7)	−0.0040 (8)	−0.0042 (8)
O2	0.0547 (10)	0.0493 (9)	0.0533 (10)	−0.0031 (8)	0.0010 (8)	−0.0097 (8)
O3	0.0334 (9)	0.0503 (9)	0.0741 (12)	−0.0118 (7)	0.0018 (8)	−0.0018 (8)
O4	0.0548 (12)	0.0705 (12)	0.0998 (15)	−0.0224 (10)	0.0286 (11)	−0.0036 (11)
O5	0.0424 (10)	0.0716 (11)	0.0606 (10)	−0.0096 (9)	−0.0145 (8)	−0.0055 (9)
Cl	0.0911 (8)	0.430 (3)	0.0770 (6)	−0.0500 (13)	−0.0245 (6)	0.0210 (12)

### Geometric parameters (Å, º)

**Table:**

C1—O3	1.473 (3)	C13—H13A	0.9700
C1—C2	1.476 (3)	C13—H13B	0.9700
C1—C10	1.531 (3)	C14—H14A	0.9600
C1—H1	0.9800	C14—H14B	0.9600
C2—O5	1.436 (3)	C14—H14C	0.9600
C2—C3	1.461 (3)	C15—H15A	0.9600
C2—H2	0.9800	C15—H15B	0.9600
C3—O5	1.444 (3)	C15—H15C	0.9600
C3—C4	1.502 (4)	C16—N1	1.461 (3)
C3—C14	1.503 (3)	C16—C17	1.502 (4)
C4—C5	1.530 (3)	C16—H16A	0.9700
C4—H4A	0.9700	C16—H16B	0.9700
C4—H4B	0.9700	C17—N2	1.450 (3)
C5—C6	1.500 (3)	C17—H17A	0.9700
C5—H5A	0.9700	C17—H17B	0.9700
C5—H5B	0.9700	C18—N2	1.460 (4)
C6—O1	1.449 (3)	C18—C19	1.500 (4)
C6—C7	1.478 (3)	C18—H18A	0.9700
C6—H6	0.9800	C18—H18B	0.9700
C7—O1	1.443 (3)	C19—N1	1.458 (3)
C7—C15	1.508 (3)	C19—H19A	0.9700
C7—C8	1.516 (3)	C19—H19B	0.9700
C8—O2	1.409 (3)	C20—C21	1.379 (5)
C8—C9	1.540 (3)	C20—C25	1.393 (5)
C8—H8	0.9800	C20—N2	1.409 (4)
C9—C10	1.545 (3)	C21—C22	1.386 (5)
C9—H9A	0.9700	C21—H21	0.9300
C9—H9B	0.9700	C22—C23	1.353 (7)
C10—C11	1.526 (3)	C22—H22	0.9300
C10—H10	0.9800	C23—C24	1.350 (8)
C11—C12	1.501 (4)	C23—Cl	1.737 (4)
C11—C13	1.520 (3)	C24—C25	1.386 (6)
C11—H11	0.9800	C24—H24	0.9300
C12—O4	1.199 (3)	C25—H25	0.9300
C12—O3	1.335 (3)	O2—H2A	0.8200
C13—N1	1.465 (3)		
			
O3—C1—C2	106.49 (17)	N1—C13—H13A	108.9
O3—C1—C10	104.81 (17)	C11—C13—H13A	108.9
C2—C1—C10	112.58 (17)	N1—C13—H13B	108.9
O3—C1—H1	110.9	C11—C13—H13B	108.9
C2—C1—H1	110.9	H13A—C13—H13B	107.7
C10—C1—H1	110.9	C3—C14—H14A	109.5
O5—C2—C3	59.81 (14)	C3—C14—H14B	109.5
O5—C2—C1	119.30 (18)	H14A—C14—H14B	109.5
C3—C2—C1	125.7 (2)	C3—C14—H14C	109.5
O5—C2—H2	113.8	H14A—C14—H14C	109.5
C3—C2—H2	113.8	H14B—C14—H14C	109.5
C1—C2—H2	113.8	C7—C15—H15A	109.5
O5—C3—C2	59.26 (14)	C7—C15—H15B	109.5
O5—C3—C4	114.4 (2)	H15A—C15—H15B	109.5
C2—C3—C4	115.1 (2)	C7—C15—H15C	109.5
O5—C3—C14	113.6 (2)	H15A—C15—H15C	109.5
C2—C3—C14	123.9 (2)	H15B—C15—H15C	109.5
C4—C3—C14	116.8 (2)	N1—C16—C17	110.8 (2)
C3—C4—C5	113.5 (2)	N1—C16—H16A	109.5
C3—C4—H4A	108.9	C17—C16—H16A	109.5
C5—C4—H4A	108.9	N1—C16—H16B	109.5
C3—C4—H4B	108.9	C17—C16—H16B	109.5
C5—C4—H4B	108.9	H16A—C16—H16B	108.1
H4A—C4—H4B	107.7	N2—C17—C16	111.8 (2)
C6—C5—C4	113.5 (2)	N2—C17—H17A	109.3
C6—C5—H5A	108.9	C16—C17—H17A	109.3
C4—C5—H5A	108.9	N2—C17—H17B	109.3
C6—C5—H5B	108.9	C16—C17—H17B	109.3
C4—C5—H5B	108.9	H17A—C17—H17B	107.9
H5A—C5—H5B	107.7	N2—C18—C19	111.9 (2)
O1—C6—C7	59.07 (14)	N2—C18—H18A	109.2
O1—C6—C5	117.00 (19)	C19—C18—H18A	109.2
C7—C6—C5	124.9 (2)	N2—C18—H18B	109.2
O1—C6—H6	114.7	C19—C18—H18B	109.2
C7—C6—H6	114.7	H18A—C18—H18B	107.9
C5—C6—H6	114.7	N1—C19—C18	111.2 (2)
O1—C7—C6	59.48 (13)	N1—C19—H19A	109.4
O1—C7—C15	113.19 (19)	C18—C19—H19A	109.4
C6—C7—C15	122.9 (2)	N1—C19—H19B	109.4
O1—C7—C8	117.09 (19)	C18—C19—H19B	109.4
C6—C7—C8	121.41 (19)	H19A—C19—H19B	108.0
C15—C7—C8	111.7 (2)	C21—C20—C25	116.9 (3)
O2—C8—C7	110.80 (18)	C21—C20—N2	121.2 (3)
O2—C8—C9	112.11 (18)	C25—C20—N2	121.9 (3)
C7—C8—C9	111.63 (18)	C20—C21—C22	121.1 (4)
O2—C8—H8	107.3	C20—C21—H21	119.4
C7—C8—H8	107.3	C22—C21—H21	119.4
C9—C8—H8	107.3	C23—C22—C21	120.6 (5)
C8—C9—C10	114.54 (17)	C23—C22—H22	119.7
C8—C9—H9A	108.6	C21—C22—H22	119.7
C10—C9—H9A	108.6	C24—C23—C22	120.0 (4)
C8—C9—H9B	108.6	C24—C23—Cl	120.2 (5)
C10—C9—H9B	108.6	C22—C23—Cl	119.7 (6)
H9A—C9—H9B	107.6	C23—C24—C25	120.3 (5)
C11—C10—C1	101.98 (17)	C23—C24—H24	119.8
C11—C10—C9	116.96 (19)	C25—C24—H24	119.8
C1—C10—C9	114.46 (18)	C24—C25—C20	121.1 (5)
C11—C10—H10	107.6	C24—C25—H25	119.5
C1—C10—H10	107.6	C20—C25—H25	119.5
C9—C10—H10	107.6	C19—N1—C16	107.29 (19)
C12—C11—C13	110.6 (2)	C19—N1—C13	109.5 (2)
C12—C11—C10	102.57 (19)	C16—N1—C13	111.6 (2)
C13—C11—C10	116.78 (19)	C20—N2—C17	116.2 (2)
C12—C11—H11	108.9	C20—N2—C18	116.1 (2)
C13—C11—H11	108.9	C17—N2—C18	111.7 (2)
C10—C11—H11	108.9	C7—O1—C6	61.45 (14)
O4—C12—O3	120.6 (2)	C8—O2—H2A	109.5
O4—C12—C11	128.6 (3)	C12—O3—C1	110.04 (18)
O3—C12—C11	110.8 (2)	C2—O5—C3	60.93 (14)
N1—C13—C11	113.45 (19)		

### Hydrogen-bond geometry (Å, º)

**Table:**

*D*—H···*A*	*D*—H	H···*A*	*D*···*A*	*D*—H···*A*
O2—H2*A*···N1	0.82	2.14	2.943 (2)	166
C1—H1···O4^i^	0.98	2.37	3.271 (3)	152
C10—H10···O1^ii^	0.98	2.41	3.329 (3)	153

Symmetry codes: (i) *x*−1/2, −*y*+1/2, −*z*+2; (ii) *x*+1/2, −*y*−1/2, −*z*+2.
